# Psychosocial Functioning, Quality of Life, Depressive Symptoms, and Meaning in Life in Older Adults with Multiple Myeloma: A Cross-Sectional Study

**DOI:** 10.3390/ijerph23030289

**Published:** 2026-02-26

**Authors:** Érika Arantes Oliveira-Cardoso, Virginia Comazzetto Tabuzo, Vitória Fernandes Andreossi, Maria Antônia Souza Oliveira, Jorge Henrique Corrêa Santos, Lucas Santos Lotério, Pedro Manoel Marques Garibaldi, Maria Isabel Ayrosa Madeira, Manoel Antônio Santos

**Affiliations:** 1 Faculty of Philosophy, Sciences and Letters of Ribeirão Preto, University of São Paulo, Ribeirão Preto 14040-901, Brazil; virginia.tabuzo@usp.br (V.C.T.); viandreossi@usp.br (V.F.A.); msouzaoliveira60@usp.br (M.A.S.O.); correasantosjh@gmail.com (J.H.C.S.); loteriolucas@gmail.com (L.S.L.); masantos@ffclrp.usp.br (M.A.S.); 2 Hemocentro Hospital, University of São Paulo, Ribeirão Preto 14051-140, Brazil; pedrogaribaldi@hotmail.com (P.M.M.G.); belayrosa@hcrp.usp.br (M.I.A.M.)

**Keywords:** multiple myeloma, quality of life, aging, depressive symptoms, meaning in life

## Abstract

**Highlights:**

**Public health relevance—How does this work relate to a public health issue?**

- Population aging and prolonged survival in multiple myeloma have expanded the cohort of older adults managing enduring physical, psychological, and social challenges.
- Understanding quality of life, depressive symptoms, and existential resources in this population addresses a growing public health need for comprehensive survivorship care.

**Public health significance—Why is this work of significance to public health?**

- Fatigue, pain, and depressive symptoms emerged as key determinants of diminished quality of life, even among clinically stable older adults.
- The presence of meaning in life emerged as a protective factor, reinforcing the importance of psycho-social and existential dimensions in public health–oriented oncology care.

**Public health implications—What are the key implications or messages for practitioners, policy makers and/or researchers in public health?**

- Multidimensional care models integrating symptom management, mental health screening, and meaning-centered interventions are essential to optimize quality of life in older adults with cancer.
- Public health policies and clinical guidelines should incorporate psychosocial and existential support as core components of geriatric oncology and chronic cancer care.

**Abstract:**

(1) Background: Older adults with multiple myeloma (MM) contend with multifaceted physical, psychological, and existential challenges that may compromise quality of life (QoL). This study examined associations between physical symptom burden, functional capacity, depression severity, meaning in life, and QoL in older adults with MM. (2) Methods: Fifty outpatients (mean age = 67.7 ± 8.8 years) completed the EORTC QLQ-C30, PHQ-9, and Meaning in Life Questionnaire (MLQ). QoL was the primary outcome. Secondary outcomes included physical symptom burden (fatigue and pain), functioning domains, depression severity, and meaning in life. Internal consistency of all instruments was satisfactory (Cronbach’s α > 0.70). Analysis and associations among variables were done using Pearson’s and Spearman’s correlation coefficients (*p* < 0.05). (3) Results: Participants reported high global QoL and preserved functioning. Fatigue and pain were most strongly associated with lower global QoL. Greater depression severeness, although mild, was correlated with higher physical symptom burden and poorer functioning. Higher Presence of Meaning was related to better QoL and lower depression severity, whereas Search for Meaning showed no significant associations. Women reported greater physical symptom burden than men. (4) Conclusions: In older adults with MM, QoL appears to be linked to interrelated physical, psychological, functional, and existential factors. Fatigue, pain, depression severity, and established meaning in life were identified as clinically relevant correlates, highlighting targets for integrated supportive care.

## 1. Introduction

Hematologic malignancies comprise a heterogeneous group of cancers arising from blood progenitor cells. Among these, multiple myeloma (MM) is particularly notable for its complex clinical manifestations. MM is characterized by the clonal proliferation of plasma cells within the bone marrow, which leads to bone injuries, anemia, immunosuppression, and renal dysfunction [1]. The median age range at diagnosis is roughly 70 years and accounts for approximately 10% of all hematologic malignancies [2]. Despite advances in therapeutic approaches that have significantly improved survival, MM continues to exert profound physical, psychological, and social consequences for patients [3].

Following diagnosis, individuals with MM commonly experience persistent pain, severe fatigue, and sleep disturbances. These symptoms frequently co-occur as symptom clusters—including psychological symptoms, pain–sleep–dry mouth cluster, and fatigue—each of which independently diminishes quality of life [4]. Pain and fatigue are closely related to psychological distress, including anxiety and depression, creating a bidirectional cycle in which worsening physical symptoms exacerbate emotional suffering, which in turn intensifies symptom perception [4,5]. High levels of distress, depression, and anxiety have been reported across all stages of the treatment, underscoring the widespread nature of psychosocial challenges faced by them [3,6].

Additional factors like limited understanding or acceptance of the prognosis, personal and financial concerns, as well as family strain and anhedonia, emerged as common stressors that added a burden to MM [7,8]. Evidence suggests that relying solely on distress screening surveys may overlook high-risk patients, thereby missing opportunities to identify socially vulnerable individuals who could benefit from additional supportive services [9]. These findings highlight gaps in comprehensive care, demanding fairer approaches to psychosocial assessment and intervention.

Pain remains a central determinant of quality of life in MM. Ludwig et al. (2021) [10] demonstrated that higher levels of self-reported pain are associated with significant reductions in health-related quality of life, including marked impairments in physical, social, and emotional functioning. Considering the broad impact of the disease, many studies have examined its relationship with depression and anxiety, showing that sociodemographic and clinical factors—such as gender, comorbidities, and hospitalization—influ1ence vulnerability to psychological distress [11,12,13]. Ribeiro-Carvalho et al. (2020) [13] reported that women were more likely to experience depression. Generally, it increases along with comorbidities and is associated with longer hospital stays.

Evidence also highlights the importance of protective factors. Positive social support, for instance, plays a relevant role, fosters emotional and social functioning, and reinforces the value of structured psycho-oncological interventions [14]. Moreover, existential and spiritual dimensions—particularly the presence of meaning in life—have emerged as powerful resources that buffer distress, reduce death-related anxiety, promote adaptive coping, and enhance psychological well-being [15]. Qualitative studies further indicate that acceptance of the disease and its prognosis are promoted by supportive relationships, positive coping strategies, and less clinical burden. On the other hand, uncontrolled disease-related manifestations may undermine adaptive adjustment [7].

Despite these insights, few studies have comprehensively examined how clinical, emotional, existential, and sociodemographic factors interact to influence quality of life, particularly in older adults with multiple myeloma (MM), who face compounded vulnerabilities related to aging and disease burden.

The primary objective of the present study is to examine the associations between global quality of life, physical symptom burden, functional capacity, and depression severity in older adults living with MM. Secondary objectives were to investigate the role of existential dimensions of meaning in life, to explore gender differences in symptom burden and psychosocial outcomes, and to assess how functional, emotional, cognitive, and social domains relate to overall quality of life.

By examining the interconnections among these clinical, psychological, existential, and sociodemographic factors, this study aims to provide an integrated understanding of the risk and protective mechanisms shaping well-being in this population. This knowledge can provide multidisciplinary strategies to enhance quality of life and optimize integrated care for older adults with MM.

**Hypotheses**

**H1.**  *Greater physical symptom burden, operationalized as higher scores on measures of pain, fatigue, dyspnea, and insomnia, will be associated with lower global quality of life.*

**H2.**  *Lower physical, emotional, cognitive, and social functioning will be associated with poorer overall quality of life.*

**H3.**  *Higher depression severity scores (PHQ-9) will be associated with lower quality of life and greater physical symptom burden.*

**H4.**  *Women will report greater physical symptom burden and lower functional outcomes than men.*

**H5.**  *Higher Presence of Meaning scores (MLQ) will be positively associated with quality of life and negatively associated with depression severity.*

**H6.**  *Search for Meaning scores (MLQ) will not show any significant association with quality of life or depression severity.*

## 2. Materials and Methods

### 2.1. Study Design

This study employed a prospective, cross-sectional observational design to examine the associations between quality of life, functional status, emotional and physical symptom burden, existential factors, and sociodemographic characteristics in older adults with multiple myeloma. Data were collected specifically for this research.

### 2.2. Participants

The study included a convenience sample of 50 older adults diagnosed with MM who were receiving outpatient care at a public oncology service in Hemocentro Hospital, in Ribeirão Preto, São Paulo, Brazil, between November 2024 and January 2025. During this period, all patients who met the criteria were invited to participate. Four patients declined. Recruitment was concluded after three consecutive outpatient clinic days in which no additional eligible patients were identified.

### 2.3. Data Collection

To assess psychological distress and quality of life, the following validated instruments were used:

The European Organization for Research and Treatment of Cancer Quality of Life Questionnaire (EORTC QLQ-C30)—European Organization for Research and Treatment of Cancer Quality of Life Questionnaire (EORTC QLQ-C30)—a widely used assessment instrument on this subject. The questionnaire includes functional scales, symptom scales, and a global health status/quality of life scale. In Brazilian validation studies, the EORTC QLQ-C30 demonstrated adequate internal consistency, with Cronbach’s alpha coefficients ranging from 0.72 to 0.86 [16].

The Patient Health Questionnaire-9 (PHQ-9)—a screening tool designed to identify the presence and severity of depressive disorders based on nine items derived from DSM-IV diagnostic criteria for major depressive disorder. The Brazilian version of the PHQ-9 has demonstrated adequate validity and reliability. In validation studies conducted in Brazil, the Hamilton Depression Rating Scale (HAM-D) was used as the gold standard to evaluate the screening performance of the PHQ-9, revealing a strong and statistically significant correlation between the instruments (ρ = 0.667, *p* < 0.001), supporting the reliability and validity of the PHQ-9 for screening depressive symptoms in the Brazilian context [17].

The Life Meaning Questionnaire—MLQ (MLQ-QSV)—assesses meaning in life through two dimensions: the Presence of Meaning and the Search for Meaning. The Brazilian version demonstrated adequate factorial validity, supporting the original two-factor structure, as well as good internal consistency, with Cronbach’s alpha coefficients of 0.85 (Presence of Meaning) and 0.89 (Search for Meaning). Evidence of convergent validity was observed through correlations with the Purpose in Life Test and the Ontological Time Perception Scale, supporting its use in the Brazilian context [18].

The Socio-demographic and Clinical Questionnaire includes data concerning: age, sex, marital status, educational level, occupation, religion, time since diagnosis, treatments received, presence of low back pain, and medication use.

### 2.4. Data Analysis

Scoring have followed official instrument guidelines.

EORTC QLQ-C30: Functional and global health scores indicate better quality of life with higher values; symptom scores indicate greater severity with higher values. Symptom interpretation: absent/mild (0–20), mild to moderate (21–40), moderate (41–60), intense (61–80), very intense (81–100). Functional scales: very poor (0–20), poor (21–40), moderate (41–60), good (61–80), excellent (81–100).

PHQ-9: Total score sum of nine items; higher scores indicate greater depression severity, categorized as minimal/none (0–4), mild (5–9), moderate (10–14), moderately severe (15–19), and severe (20–27). MLQ-QSV: Two subscales scored separately. Higher Presence of Meaning scores indicate a stronger perceived sense of life meaning; higher Search of Meaning scores indicate greater engagement in the pursuit of purpose and life goals. Score interpretation: very low (5–14), low (15–19), moderate (20–24), high (25–29), very high (30–35).

The socio-demographic and clinical variables were summarized using frequencies, percentages, means, and standard deviations. All scoring procedures followed standardized guidelines to ensure consistency, transparency, and interpretability.

### 2.5. Statistical Analysis

Analyses were conducted using SPSS version 20.0. Descriptive statistics were used to summarize the demographic and clinical characteristics of the sampling. The consistency of the instruments in the present sample was assessed using Cronbach’s alpha coefficients. Associations between numerical variables were assessed using Spearman’s correlation coefficient. Differences in scale scores between participants who practiced religion and those who did not, as well as between men and women, were analyzed using the Mann–Whitney U test. To determine which group exhibited greater severity on the assessed constructs, Mean Rank values from the Mann–Whitney U test were examined, with higher Mean Ranks indicating greater severity or burden as measured by the respective scales. Relationships between categorical variables, such as meaning in life and religious practice, were evaluated using the chi-square test of independence (χ^2^). Categories with small cell counts were combined when necessary to ensure statistical validity. The significance level was set at *p* < 0.05.

### 2.6. Ethical Aspects

This study was approved by the Institutional Review Board and conducted in conformity with Resolution No. 466/12, establishing ethical guidelines for research with human subjects. All participants were informed about the study objectives, procedures, and confidentiality conditions, and only those who consented previously were included. About the online interviewed participants, the consentment have being given verbally and formally recorded. Confidentiality was ensured throughout the study.

### 2.7. Researcher Bias and Data Rigor

To minimize potential researcher bias in this quantitative study, the research instruments were carefully selected and applied according to standardized guidelines. Data collection procedures were standardized, and all participants received the same instructions to ensure consistency. The data entry and analysis processes were double-checked for accuracy. Statistical analyses were conducted following established protocols to ensure objective interpretation of the results. Any deviations from the planned procedures were documented to maintain transparency and rigor in the research process.

## 3. Results

### 3.1. Sample Characteristics

The average participants’ age were 67.7 years old (SD = 8.8) and the gender distribution were equivalent for both men and women (50%). The average time since diagnosis was by 4.5 years (SD = 4.71), indicating a predominantly chronic disease trajectory.

Effectively, participants’ general performance status was preserved, as reflected by the Karnofsky Performance Status Index (meaning = 81.79, SD = 7.58). Ten of them had previously undergone the autologous hematopoietic stem cell transplantation (ASCT), and 27 participants presented low back pain. Sociodemographically, most of the participants identified themselves as White and Catholic, had an elementary education degree, were married, and also retired or unemployed. Their average monthly income were about two minimum wages. Detailed demographic and clinical characteristics are set in Table 1.

It was mandatory that participants could understand the study procedures, standards and provide reliable self-reports, to take part of this study. The ones who were not oriented or had cognitive impairments that hindered accurate self-reporting were excluded.

Internal consistency was assessed for all instruments and found to be acceptable (Cronbach’s α ≥ 0.70). All EORTC QLQ-C30 subscales met this criterion. The PHQ-9 demonstrated good reliability (α= 0.80), and the MLQ subscales showed acceptable internal consistency (Presence α = 0.70; Search α = 0.85).

Descriptive statistics for the EORTC QLQ-C30 domains are presented in Table 2. The table includes mean scores, standard deviations (SD), medians, and interquartile ranges (IQR) for all functioning and symptom-related domains.

### 3.2. Depression

Table 3 presents the meanings, the standard deviation (SD), and the median (interquartile range, IQR) for each PHQ-9 item and for the total score among the 50 patients.

Participants were categorized according to standard PHQ-9 severity thresholds. The distribution was as follows: 70% minimal to mild, 26% moderate to moderately severe, and 4% severe.

### 3.3. Meaning in Life Questionnaire (MLQ)

Table 4 presents descriptive statistics for each of the 10 MLQ items, as well as for the two dimensions: Presence of Meaning and Search for Meaning.

### 3.4. Associations Among Study Variables

Descriptive statistics and Mann–Whitney U tests were conducted to compare symptom-related domains between men and women. Statistically significant differences were observed for nausea and vomiting, appetite loss, and constipation. Meaning ranks and scores for these domains are presented in Table 5.

Mann–Whitney U tests were conducted to compare insomnia severeness and meaning in life dimensions among religious practitioners and non-practitioners. A statistically substancial difference was observed for insomnia severeness (U = 126.00, *p* = 0.024), where non-practitioners showing higher meaning scores (M = 3.00, SD = 1.61; Mean Rank = 33.55) than practitioners (M = 1.82, SD = 1.32; Mean Rank = 23.23).

For the Presence of Meaning dimension of the MLQ, a statistically considerable difference was found (U = 331.0, *p* = 0.003), where practitioners exhibited higher meaning ranks (28.49) than non-practitioners (14.91). Non-significant difference was observed for the Search for Meaning dimension (U = 286.5, *p* = 0.086). Table 6 presents descriptive statistics for the following variables, where groups performed unlikely between themselves.

Spearman’s rank-order correlations were calculated to examine relationships among depression severity (PHQ-9 total score), global quality of life (EORTC QLQ-C30), and meaning in life dimensions (MLQ). Statistically significant associations (*p* < 0.05) are summarized in Table 7.

Higher PHQ-9 scores were significantly associated with greater symptom burden, particularly insomnia, fatigue, dyspnea, pain, nausea, and diarrhea. In addition, higher depression severeness was correlated with lower cognitive, emotional, personal role, physical, and social functioning scores, as well as lower levels of presence of meaning in life.

Meanwhile, global quality-of-life scores endorsed positive correlations with physical, personal role, emotional, cognitive, social functioning, and presence of meaning, while pain was inversely associated with global quality of life. The search for meaning dimension did not show significant associations with quality of life or depression severeness.

These findings underscore the multidimensional interplay among symptom burden, functional status, depressive severity, and existential resources in shaping perceived well-being among older adults with multiple myeloma.

## 4. Discussion

This study examined quality of life (QoL) in a well-defined sampling of older adults (≥60 years) living with long-standing multiple myeloma (MM), a population profile that remains underrepresented in oncology research. Overall, participants reported relatively preserved physical, emotional, cognitive, and social functioning, as well as favorable global health status. The findings indicate that older adults may report sustained functional capacity and psychosocial adjustments despite persistent disease-related symptoms under a clinical context. By focusing specifically on long-standing MM in later life, this study contributes to a distinct approach to understanding QoL regarding aging, chronic disease exposure, and survivorship.

Putting the present study in perspective and taking international data found into account, it reveals similarities and distinctions as well. The Global Health Status/QoL scores’ sampling (75.17 ± 24.37) was higher than the one reported in a multicentered British German study (about 60) [19]. Alike, QoL scores exceeded baseline values observed in phase III ALCYONE trial (50–52), which includes just diagnosed transplant-ineligible patients [20]. These differences may reflect variations in the stage of the disease, the treatment timing, and patient’s characteristics. Individuals living with long-standing MM may have undergone psychological and functional adaptation processes, once newly diagnosed patients are often assessed during more symptomatic or treatment-intensive phases.

About the functional domains, personal role and social functioning, they were relatively preserved, suggesting sustained in daily responsibilities and interpersonal relationships engagement. Physical and emotional functioning were slightly lower, possibly reflecting the cumulative disease burden and psychosocial variability across populations [19]. These findings underline the relevance of continuous functional assessment in older adults with MM, where even global QoL appears favorable.

Pain and fatigue emerged as the most prominent symptoms, despite a generally modest overall symptom burden. Levels of tiredness were lower than those reported in the ALCYONE trial (~32 vs. ~41–42), where pain scores were slightly higher than in the European cohort but comparable to just MM diagnosed populations [20]. These findings are consistent with previous research highlights concerning pain and exhaustion as central features throughout the MM treatment trajectory [3,5]. Prior studies also document strong associations between physical discomfort and emotional distress—both domains are closely interconnected [4]. In our sample, greater symptom severeness was linked to lower QoL scores, emphasizing systematic symptom monitoring and timely supportive interventions.

Insomnia scores were comparable to those observed in other cohorts (~25–30), whereas financial difficulties were more pronounced. This finding may reflect differences in healthcare systems and socioeconomic support structures [20] and is consistent with growing evidence on financial toxicity in MM. Routine assessment of financial strain and social vulnerabilities may be relevant as a component of comprehensive patient-centered care.

When compared with Brazilian cohorts, evaluated before and after autologous stem cell transplantation (ASCT) findings can be contextualized even further. Global health status and personal role, social, and cognitive functioning parameters were like previously reported Brazilian data [21], supporting the observation of relatively preserved functioning in this population. Instead, symptoms such as pain, fatigue, dyspnea, and constipation were more pronounced, possibly reflecting the combined effects of aging and long-standing disease. Nausea, appetite loss, and diarrhea remained low, and insomnia and financial difficulties were comparable to prior national findings [22].

Depressive symptoms were mild on average but showed significant associations with poorer overall functioning and greater physical symptom burden. While most participants reported occasionally depressive manifestations, and also suicidal ideation was rare, a subset presented moderate to severe symptoms. These findings are consistent with prior studies demonstrating that even subclinical depressive symptoms are associated with lower health-related QoL in oncological populations [11,13,23]. The results support clinical routine relevance, particularly in older adults who may present subtle or overlapping somatic symptoms following up a MM psychological screening.

A key contribution of this study is its exploratory examination of existential dimensions, particularly meaning in life, as correlates of psychological well-being in this population. Participants who reported to have religious practice also demonstrated higher scores in the Presence of Meaning dimension. These results are consistent with emerging evidence suggesting that religiosity may influence psychological and behavioral health outcomes through pathways involving reduced anxiety and depressive symptoms, as well as broader psychosocial resources [24,25,26,27]. These findings should be interpreted cautiously and considered exploratory, once the cross-sectional design precludes causal inference.

Gender-based differences were also observed. Women reported higher levels of fatigue, pain, nausea, appetite loss, and constipation, consistent with established patterns in oncological and general populations [28,29]. Men reported slightly higher dyspnea and financial distress, although these differences were modest. These findings reinforce the importance of gender-sensitive approaches to symptom assessment and individualized supportive care.

Taken together, the observed associations illustrate the interconnected nature of physical symptoms, depressive symptoms, functional status, and existential resources in shaping QoL. Rather than operating independently, these domains appear to coexist and interact within the lived experience of older adults with MM. Clinically, the findings support a multidimensional care model that incorporates systematic symptom assessment, psychological screening, attention to financial and social stressors, and recognition of existential concerns. A multidisciplinary approach integrating oncology, psychology, social work, physiotherapy, and spiritual care may enhance patient-centered management in this population.

An additional interpretative consideration concerns the relatively favorable global quality of life observed in this sampling despite the chronic and potentially life-threatening nature of multiple myeloma. This pattern may reflect adaptive processes commonly described in chronic illness, including psychological adjustment, recalibration of internal standards, and a sort of shifts in life priorities—conceptualized phenomena in the literature as “response shift” in quality-of-life research [30]. Over time, individuals living with long-standing disease may redefine well-being, leading to stable or even positive self-reported QoL despite ongoing symptom burden. Furthermore, survivorship effects may partially contribute to these findings, as patients with longer disease trajectories may represent a subgroup characterized by greater functional resilience or more favorable treatment response compared to newly diagnosed cohorts, as well as those included in the ALCYONE trial [20] and other European samples [27]. These mechanisms highlight the dynamic and subjective nature of QoL assessment and reinforce the importance of interpreting patient-reported outcomes within a broader context of illness adaptation and survivorship in older adults with MM.

Several limitations should be acknowledged. First, the modest sampling size and the focus on older adults with long-standing multiple myeloma may limit generalizability to younger individuals or those newly diagnosed. Second, the cross-sectional design captures a single point within a dynamic disease trajectory; therefore, the observed associations should be interpreted as correlational, and no conclusions regarding directionality or causality can be drawn. Third, reliance on self-report instruments may introduce reporting biases, including recall and social desirability effects. In addition, detailed clinical variables—such as disease stage (ISS/R-ISS), line of therapy, cumulative treatment exposure, comorbidity burden, and specific pain etiology—were not systematically available, limiting more granular interpretation of symptom burden and quality-of-life outcomes. Finally, religiosity was assessed throughout a single dichotomous indicator of religious practice, which does not capture the multidimensional nature of spirituality. The relatively small subgroup of non-practitioners further constrains the robustness of group comparisons. Accordingly, findings related to religious practice should be considered preliminary and a hypothesis-generator.

Future research would benefit from longitudinal designs capable of examining changes in QoL, symptom burden, depression, and existential dimensions over time. Integration of comprehensive clinical and geriatric assessments, as well as qualitative approaches exploring meaning-making processes, may further enrich understanding on this study subject. Interventional studies evaluating psychological, existential, and multidisciplinary geriatric oncology programs are warranted to determine whether these approaches are associated with improved patient-reported outcomes in older adults with MM.

From a clinical perspective, findings support the implementation of multidimensional routine screening protocols in older adults with MM, integrating symptom assessment, depression screening, and evaluation of existential well-being. Early referral to psycho-oncology services, structured fatigue and pain management programs, financial counseling, and, when appropriate, spiritual care support may contribute to improved patient-reported outcomes. Embedding these strategies within geriatric oncology and palliative care pathways may optimize comprehensive care in this growing population.

## 5. Conclusions

The present study provides insight into the quality of life of older adults living with long-standing MM. Despite persistent symptoms such as fatigue and pain, these patients generally maintain functional capacity and report relatively favorable quality of life. Physical, emotional, and cognitive functioning, together with psychological well-being and a sense of meaning in life, are closely interrelated and shape overall quality of life. Women experience a higher symptom burden, underscoring the need for gender-sensitive care. These findings emphasize the value of a multidimensional, multidisciplinary approach to clinical management, integrating symptom monitoring, psychological support, and interventions that foster meaning and coping to optimize quality of life in this vulnerable population.

## Figures and Tables

**Table 1:** Participants’ Characterization (n = 50).

Variables	Frequency and Percentage	Meaning and SD
Sex		
Female	25 (50%)	
Male	25 (50%)	
Age (years)		Meaning = 67.7 SD = 8.8
60–65	17 (34%)	
66–70	11 (22%)	
70+	22 (44%)	
Education		
No formal education	4 (08%)	
Elementary School	30 (60%)	
High School	10 (20%)	
Higher Education	6 (12%)	
Occupation		
Formal employment	3 (06%)	
Informal work	2 (04%)	
Retiree/Unemployed	45 (90%)	
Religion		
Catholic	31 (62%)	
Other	19 (38%)	
Marital Status		
Married	29 (58%)	
Widow/Widower	13 (26%)	
Single	8 (16%)	
Average Income * Diagnosis (years)		Meaning = 4.5 SD = 4.71
1–2	21 (42%)	
3–7	19 (38%)	
7+	10 (20%)	

* Average Income (minimum wages)—the average monthly income expressed in terms of the national minimum wage. For reference, the Brazilian minimum wage in 2025 is approximately: R$1.518 per month (~€250/$270).

**Table 2:** Mean, standard deviation (SD), and median scores for EORTC QLQ-C30 domains (n = 50).

Domain	Meaning and SD	Median and IQR (Q1–Q3)
Global Health status	75.17 (24.37)	79.15 (66.70–100.00)
Functioning		
Physical functioning	62.66 (21.49)	60.00 (46.70–80.00)
Role functioning	73.32(32.65)	83.30 (66.70–100.00)
Emotional functioning	69.00 (30.19)	75.00 (52.08–100.00)
Cognitive functioning	74.76 (29.66)	83.30 (50.00–100.00)
Social functioning	73.67 (32.16)	83.33 (54.18–100.00)
Symptom-related		
Fatigue	32.73 (28.91)	22.00 (11.00–44.30)
Nausea and vomiting	10.00 (17.81)	0.00 (0.00–16.70)
Pain	38.67 (34.57)	33.30 (16.70–62.35)
Dyspnoea	15.99 (26.29)	0.00 (0.00–33.30)
Insomnia	28.00 (35.87)	0.00 (0.00–58.35)
Appetite loss	13.33 (28.57)	0.00 (0.00–0.00)
Constipation	29.33 (37.89)	0.00 (0.00–66.70)
Diarrhoea	10.66 (20.68)	0.00 (0.00–24.80)
Financial difficulties	32.00 (37.48)	16.65 (0.00–66.70)

**Table 3:** Summary of depression scores (PHQ-9) among 50 patients.

Item	Meaning and SD	Median
Q1—Little interest or pleasure in doing things	0.90 (1.07)	1.00 (0.00–1.00)
Q2—Feeling down, depressed, or hopeless	0.94 (1.02)	1.00 (0.00–2.00)
Q3—Trouble falling or staying asleep, or sleeping too much	1.20 (1.25)	1.00 (0.00–2.00)
Q4—Feeling tired or having little energy	1.20 (1.18)	1.00 (0.00–2.00)
Q5—Poor appetite or overeating	1.16 (1.22)	1.00 (0.00–2.00)
Q6—Feeling bad about yourself, or that you are a failure	0.70 (1.04)	0.00 (0.00–1.00)
Q7—Trouble concentrating on things	0.74 (1.14)	0.00 (0.00–1.75)
Q8—Moving or speaking slowly, or being fidgety/restless	0.74 (1.01)	0.00 (0.00–1.00)
Q9—Thoughts that you would be better off dead	0.14 (0.45)	0.00 (0.00–0.00)
Total PHQ-9 score	7.72 (5.94)	6.00 (3.00–11.75)

**Table 4:** Descriptive statistics of the Meaning in Life Questionnaire (MLQ) items.

Item	Meaning and SD	Median (IQR)
Q1	5.90 (1.82)	7.00 (6.00–7.00)
Q2	4.32 (2.53)	5.00 (1.00–7.00)
Q3	4.72 (2.44)	5.50 (2.00–7.00)
Q4	5.92 (1.70)	7.00 (5.25–7.00)
Q5	5.94 (1.88)	7.00 (6.00–7.00)
Q6	5.58 (1.91)	6.00 (5.00–7.00)
Q7	4.66 (2.50)	6.00 (1.25–7.00)
Q8	4.32 (2.54)	5.00 (1.00–7.00)
Q9	3.68 (2.64)	3.00 (1.00–7.00)
Q10	4.46 (2.38)	5.50 (2.00–6.75)
Dimension		
Presence of Meaning	27.02 (6.68)	29.00 (25.25–30.75)
Search for Meaning	22.48 (9.90)	24.50 (15.25–31.00)

**Table 5:** Descriptive statistics for symptom-related domains with statistically significant differences between men and women.

Variable	Group	N	Meaning	SD	SE	Coefficient of Variation	Mean Rank	Sum of Ranks
Nausea and Vomiting	Men	25	1.16	0.47	0.10	0.41	22.42	560.50
	Women	25	1.56	0.87	0.17	0.56	28.58	714.50
Appetite Loss	Men	25	1.16	0.80	0.16	0.69	21.18	529.50
	Women	25	1.84	1.28	0.26	0.70	29.82	745.50
Constipation	Men	25	1.52	1.01	0.20	0.66	20.36	509.00
	Women	25	2.80	1.73	0.35	0.62	30.64	766.00

**Table 6:** Descriptive statistics for symptom-related and meaning-in-life variables showing statistically significant differences between religious practitioners and non-practitioners.

Variable	Group	N	Mean	SD	SE	Coefficient of Variation	Mean Rank	Sum of Ranks
Insomnia	Practitioners	39	1.82	1.32	0.21	0.72	23.23	906.00
	Non-practitioners	11	3.00	1.61	0.49	0.54	33.55	369.00
Presence of Meaning	Practitioners	39	4.05	1.09	0.176	0.271	28.49	1111.00
	Non-practitioners	11	3.18	0.98	0.296	0.30	14.91	164.00

**Table 7:** Correlations among quality of life, depressive severity and meaning in life.

Variables correlated	R	*p*-Value	Interpretation
PHQ-9 × Insomnia	0.55	<0.01	Moderate positive
PHQ-9 × Fatigue	0.50	<0.01	Moderate positive
PHQ-9 × Dyspnea	0.48	<0.01	Moderate positive
PHQ-9 × Pain	0.46	<0.01	Moderate positive
PHQ-9 × Nausea	0.32	<0.05	Weak positive
PHQ-9 × Diarrhea	0.30	<0.05	Weak positive
PHQ-9 × Cognitive Function	−0.58	<0.01	Strong negative
PHQ-9 × Emotional Function	−0.50	<0.01	Moderate negative
PHQ-9 × Role Function	−0.48	<0.01	Moderate negative
PHQ-9 × Physical Function	−0.45	<0.01	Moderate negative
PHQ-9 × Social Function	−0.42	<0.01	Moderate negative
PHQ-9 × Global Quality of Life	−0.40	<0.01	Moderate negative
PHQ-9 × Presence of Meaning	−0.46	<0.01	Moderate negative
Global QoL × Physical Function	0.48	<0.01	Moderate positive
Global QoL × Role Function	0.45	<0.01	Moderate positive
Global QoL × Emotional Function	0.35	<0.05	Weak positive
Global QoL × Cognitive Function	0.32	<0.05	Weak positive
Global QoL × Social Function	0.50	<0.01	Moderate positive
Global QoL × Pain	−0.28	<0.05	Weak negative
Global QoL × Presence of Meaning	0.28	<0.05	Weak positive

## Data Availability

The data presented in this study are available on request from the corresponding author. The data are not publicly available due to privacy or ethical restrictions.

## Abbreviations

The following abbreviations are used in this manuscript:

MM	Multiple Myeloma
QoL	Quality of Life
SD	Standard Deviation
EORTC QLQ-C30	European Organization for Research and Treatment of Cancer Quality of Life Questionnaire
PHQ-9	Patient Health Questionnaire-9
MLQ-QSV	Meaning in Life Questionnaire—MLQ

## References

[1] Guedes A., Becker R.G., Teixeira L.E.M. (2023). Multiple Myeloma (Part 1): Update on Epidemiology, Diagnostic Criteria, Systemic Treatment and Prognosis. Rev. Bras. Ortop..

[2] Zhuge L., Lin X., Fan Z., Jia M., Lin C., Zhu M., Teng H., Chen G. (2025). Global, Regional and National Epidemiological Trends of Multiple Myeloma from 1990 to 2021: A Systematic Analysis of the Global Burden of Disease Study 2021. Front. Public Health.

[3] Suzuki N., Okuyama T., Akechi T., Kasumoto S., Ri M., Inagaki A., Kayukawa S., Yano H., Yoshida T., Shiraga K. (2022). Symptoms and Health-Related Quality of Life in Newly Diagnosed Multiple Myeloma Patients: A Multicenter Prospective Cohort Study. Jpn. J. Clin. Oncol..

[4] Chen F.J., Leng Y., Ni J.Y., Niu T., Zhang L., Li J., Zheng Y. (2022). Symptom Clusters and Quality of Life in Outpatients with Multiple Myeloma. Support. Care Cancer.

[5] Rogers L.P.W., Rennoldson M. (2020). Are Pain and Fatigue in Multiple Myeloma Associated with Psychosocial Factors? A Systematic Review. Cancer Nurs..

[6] O’Donnell E.K., Shapiro Y.N., Yee A.J., Nadeem O., Hu B.Y., Laubach J.P., Branagan A.R., Anderson K.C., Mo C.C., Munshi N.C. (2022). Quality of Life, Psychological Distress, and Prognostic Perceptions in Patients with Multiple Myeloma. Cancer.

[7] Hermann M., Kühne F., Rohrmoser A., Preisler M., Goerling U., Letsch A. (2021). Perspectives of Patients with Multiple Myeloma on Accepting Their Prognosis: A Qualitative Interview Study. Psychooncology.

[8] Richter J., Sanchez L., Biran N., Wang C.K., Tanenbaum K., DeVincenzo V., Grunman B., Vesole D.H., Siegel D.S., Pecora A. (2021). Prevalence and Survival Impact of Self-Reported Symptom and Psychological Distress among Patients with Multiple Myeloma. Clin. Lymphoma Myeloma Leuk..

[9] Estrada-Merly N., Wu J.F., Pezzin L.E., Nataliansyah M.M., D’Souza A. (2025). Completion of Distress Screening and Correlates of Distress Score in Multiple Myeloma Patients at Initial Transplant Evaluation. Eur. J. Haematol..

[10] Ludwig H., Bailey A.L., Marongiu A., Khela K., Milligan G., Carlson K.B., Rider A., Seesaghur A. (2021). Patient-Reported Pain Severity and Health-Related Quality of Life in Patients with Multiple Myeloma in Real-World Clinical Practice. Cancer Rep..

[11] Krebber A.M.H., Buffart L.M., Kleijn G., Riepma I.C., Bree R., Leemans C.R., Becker A., Brug J., Straten A., Cuijpers P. (2014). Prevalence of Depression in Cancer Patients: A Meta-Analysis of Diagnostic Interviews and Self-Report Instruments. Psychooncology.

[12] Linden W., Vodermaier A., MacKenzie R., Greig D. (2012). Anxiety and Depression after Cancer Diagnosis: Prevalence Rates by Cancer Type, Gender, and Age. J. Affect. Disord..

[13] Ribeiro-Carvalho F., Gonçalves-Pinho M., Bergantim R., Freitas A., Fernandes L. (2020). Trend of Depression and its Association with Sociodemographic and Clinical Factors among Multiple Myeloma Hospitalizations: A Portuguese Nationwide Study from 2000 to 2015. Psychooncology.

[14] Greinacher A., Kuehl R., Mai E.K., Goldschmidt H., Wiskemann J., Fleischer A., Rasche L., Dapunt U., Maatouk I. (2024). The Impact of Different Forms of Social Support on Health-Related Quality of Life in Patients with Multiple Myeloma and Its Precursor States. J. Cancer Res. Clin. Oncol..

[15] Yang Y., Chen Y., Ou M., Gong Y., Yang R., Liu X., Xia W., Chen F., Zheng H., Xu X. (2024). The Mediating Role of Meaning in Life between Experiential Avoidance and Death Anxiety among Cancer Patients: A Cross-Sectional Study. BMC Cancer.

[16] Michels F.A.S., Latorre M.D.O., Maciel M.D.S. (2013). Validity, Reliability and Understanding of the EORTC QLQ-C30 and EORTC-BR23, Quality of Life Questionnaires Specific for Breast Cancer. Rev. Bras. Epidemiol..

[17] de Souza R., Feitosa F.B., Rodríguez T.D.M., Missiatto L.A.F. (2021). Rastreamento de sintomas de depressão em policiais penais: Estudo de validação do PHQ-9. Rev. Bras. Multidiscip..

[18] de Aquino T.A.A., Veloso V.G., de Aguiar A.A., Serafim T.D.B., Pontes A.d.M., Pereira G.d.A., Fernandes A.S. (2015). Questionário de Sentido de Vida: Evidências de sua Validade Fatorial e Consistência Interna. Psicol. Cienc. Prof..

[19] Proskorovsky I., Lewis P., Williams C.D., Jordan K., Kyriakou C., Ishak J., Davies F.E. (2014). Mapping EORTC QLQ-C30 and QLQ-MY20 to EQ-5D in patients with multiple myeloma. Health Qual. Life Outcomes.

[20] Knop S., Mateos M.-V., Dimopoulos M.A., Suzuki K., Jakubowiak A., Doyen C., Lucio P., Nagy Z., Usenko G., Pour L. (2021). Health-related quality of life in patients with newly diagnosed multiple myeloma ineligible for stem cell transplantation: Results from the randomized phase III ALCYONE trial. BMC Cancer.

[21] Malta J.S., Drummond P.L.d.M., Silveira L.P., Costa N.L., dos Santos R.M.M., Machado C.J., Reis A.M.M., de Pádua C.A.M. (2022). Efeito de regimes terapêuticos e polifarmácia na qualidade de vida relacionada à saúde de pessoas com mieloma múltiplo: Um estudo transversal em Belo Horizonte, Brasil. Curr. Med. Res. Opin..

[22] Etto L.Y., Morelli V.M., Silva V.C., Hungria V.T.M., Ciconelli R.M., Almeida M.S.S., De Oliveira J.S.R., Barros J.C., Durie B.G., Colleoni G.W.B. (2011). Transplante autólogo de células-tronco melhora a qualidade de vida de pacientes brasileiros com mieloma múltiplo em situação de vulnerabilidade socioeconômica. Clinics.

[23] Fischer J., Knop S., Danhof S., Einsele H., Keller D., Löffler C. (2022). The Influence of Baseline Characteristics, Treatment and Depression on Health-Related Quality of Life in Patients with Multiple Myeloma: A Prospective Observational Study. BMC Cancer.

[24] Berthold A., Ruch W. (2014). Satisfaction with Life and Character Strengths of Non-Religious and Religious People: It’s Practicing One’s Religion that Makes the Difference. Front. Psychol..

[25] Basis N., Boker L.K., Shochat T. (2025). Religiosity, Religious Orientation, and a Good Night’s Sleep: The Role of Anxiety and Depression. J. Sleep. Res..

[26] Hill T.D., Deangelis R., Ellison C.G. (2018). Religious Involvement as a Social Determinant of Sleep: An Initial Review and Conceptual Model. Sleep. Health.

[27] Pengpid S., Peltzer K. (2025). Religiosity and Mental and Behavioural Health among Community-Dwelling Middle-Aged and Older Adults in Thailand: Results of a Longitudinal National Survey in 2015–2020. J. Relig. Health.

[28] Cheung W.Y., Le L.W., Gagliese L., Zimmermann C. (2011). Age and Gender Differences in Symptom Intensity and Symptom Clusters among Patients with Metastatic Cancer. Support. Care Cancer.

[29] Liao J., Hu X., Wei X., Dai W., Yu H., Tian X., Wang Y., Qin Q., Xu N., Li Y. (2025). Sex-Related Differences in Postoperative Patient-Reported Outcomes among Lung Cancer Patients: A Multicenter Cohort Study. BMC Cancer.

[30] Sprangers M.A.G., Schwartz C.E. (1999). Integrating response shift into health-related quality of life research: A theoretical model. Soc. Sci. Med..

